# The diagnostic and prognostic value of magnetic resonance imaging and computed tomography parameters in non-small cell lung cancer

**DOI:** 10.12669/pjms.41.6.12071

**Published:** 2025-06

**Authors:** Lingna Bian, Jian Wang, Zhiliang Zhang, Yunyi Zhang

**Affiliations:** 1 Lingna Bian, Department of Radiology, Tongde Hospital of Zhejiang Province Afflicted to Zhejiang Chinese Medical University (Tongde hospital of Zhejiang Province), 310012, China; 2 Jian Wang, Department of Radiology, Tongde Hospital of Zhejiang Province Afflicted to Zhejiang Chinese Medical University (Tongde hospital of Zhejiang Province), 310012, China; 3 Zhiliang Zhang Department of Radiology, Zhejiang Hospital Hangzhou, 310013, P.R. China; 4 Yunyi Zhang , Department of Radiology, Tongde Hospital of Zhejiang Province Afflicted to Zhejiang Chinese Medical University (Tongde hospital of Zhejiang Province), 310012, China

**Keywords:** Computed tomography, Magnetic resonance imaging, Non-small cell lung cancer, Parameters

## Abstract

**Objective:**

To explore the diagnostic and prognostic value of magnetic resonance imaging (MRI) and computed tomography (CT) parameters in patients with non-small cell lung cancer (NSCLC).

**Methods:**

This retrospective analysis of the collected data included a cohort of 108 NSCLC patients treated in Tongde Hospital of Zhejiang Province from January 2020 to March 2022, matched in a 1:1 ratio with a cohort of 108 patients with benign lung diseases. All patients underwent MRI and CT examinations, and the MRI and CT parameters were compared between the groups, as well as between patients with different pathological features and therapeutic effects.

**Results:**

The MRI and CT parameters were significantly different between the patients with NSCLC and benign lesions and those with different pathological features and prognoses (*P*<0.05). There was a considerable correlation between MRI and CT parameters and the disease staging, lymph node metastasis (LNM), pathological type, differentiation degree, and prognosis of NSCLC (*P*<0.05).

**Conclusions:**

MRI and CT parameters have certain diagnostic and prognostic value in NSCLC and can be used to assess the disease and prognosis of this group of patients.

## INTRODUCTION

Lung cancer is associated with a high incidence of mortality and severely impacts the quality of life and physical and mental health of patients.^1^,^2^ Non-small cell lung cancer (NSCLC) is one of the most common types of lung cancer, accounting for about 80% of the total number of lung cancer cases.^2^,^3^ Early NSCLC lacks specific clinical manifestations and is usually discovered incidentally during physical examination.^3^,^4^ Therefore, most patients are diagnosed late in the course of the disease, significantly worsening treatment efficiency and prognosis.^2^–^4^

While pathological examination is considered the “gold standard” for diagnosing NSCLC, it has poor reproducibility and cannot dynamically assess changes in the condition, making it difficult to guide disease diagnosis and treatment.^2^–^5^ Magnetic resonance imaging (MRI) and computed tomography (CT) are two commonly used diagnostic methods for various malignancies.^6^,^7^ MRI allows the obtaining of multidirectional three-dimensional and tomographic images, thus providing a reference for disease diagnosis and treatment.^7^

However, lung imaging of lungs presents unique challenges due to the presence of air. MRI signals are susceptible to interference, resulting in poor visualization of subtle lung structures.^7^,^8^ In contrast, CT can present lesions’ location and relationship with surrounding tissues through multi-plane reconstruction and thin-layer CT-enhanced scanning techniques.^6^ However, it uses ion radiation and nephrotoxic contrast agents, which limits the applicability of CT for early screening in clinical practice. Additionally, CT-derived images cannot identify the pathological type of the mass.^6^,^9^

Currently, there is limited literature analyzing the correlation between MRI and MSCT examination parameters and the pathological characteristics and prognosis of NSCLC. This study aimed to clarify the diagnostic and prognostic value of MRI and CT parameters and possible correlation with different pathological features of NSCLC. The results may provide reference and practical guidance for diagnosing, treating, and evaluating NSCLC.

## METHODS

Clinical records of 108 NSCLC patients treated in Tongde Hospital of Zhejiang Province from January 2020 to March 2022 were matched in a 1:1 ratio with a cohort of 108 patients with benign lung diseases. The matching was performed based on comparable age, gender, and BMI to minimize the potential influence of these demographic factors on MRI and CT parameter measurements.

### Ethical Approval:

This study was approved by the Tongde Hospital Ethics Committee (Ref. No. 2022-029-JY, Dated: February 18, 2022). All procedures involving human subjects adhered to the 1964 Declaration of Helsinki and its subsequent amendments or equivalent ethical standards. Given the retrospective nature of the study, informed consent was waived by the Ethical Committee of Tongde Hospital of Zhejiang Province. All data were stored securely, and confidentiality was maintained throughout the study.

### Inclusion criteria:

- NSCLC group meets the diagnostic criteria for NSCLC and is confirmed by pathological examination.^1^
- The benign group had tuberculosis, inflammatory pseudotumor, pulmonary hamartoma, granuloma, and other benign lung disorders detected by pathological investigation.
- Not receiving any treatment before the examination.
- The clinical imaging data is complete.

### Exclusion criteria:

- Patients with benign or malignant tumors in other parts of the body.
- Patients with organic lesions in organs such as kidneys and liver.
- Patients with metal implants in their bodies.
- The lesions are sub-solid nodules and ground glass nodules.

### MRI:

The imaging was done using Siemens 3.0 T MRI (Siemens, Germany). The patient was assisted in achieving a supine position. The scan was done from the thoracic entrance to the diaphragm. Flat scan parameter settings were as follows: echo time of 1.6 ms, repetition time of 3.8 ms, matrix of 224 × 224, layer thickness of 8 mm, interlayer spacing of 1-1.5 mm, scan time of 16-19 s. Spin echo sequence scanning was performed after completing the flat scan, using the following parameter settings: echo time of 190 ms, repetition time of 14.3 s, matrix of 384 × 256, layer thickness of 5-7 mm, and interlayer spacing of 1-1.5 mm. T1WI and fast spin echo sequence T2WI were collected.

The relevant scanning parameters were set as follows: echo time of 90 ms, repetition time of 87.3 ms, matrix of 320 × 224, layer thickness of 4-6 mm, interlayer spacing of 1-1.5 mm, and scanning time of 120 s. Diffusion-weighted imaging (DWI) inspection was performed after completing the flat scan, and the value of b was set to 800 s/mm2. The relevant inspection images were transmitted to the workstation to generate ADC values. Dynamic contrast-enhanced MRI (DCE-MRI) inspection was performed after completing the DWI inspection. Related scanning parameter settings were as follows: scanning flip angles of 5° and 15°, echo time of 4.43 ms, repetition time of 1.5 ms, matrix of 128 × 192, and layer thickness of 3-5 mm. A contrast agent (0.2 ml/kg) was injected intravenously at a rate of 2-ml/s. Relevant data were transferred to the data processing system to avoid necrotic areas and cystic lesions. The region of interest was selected, and the extravascular extracellular volume fraction of the tissue (Ve), rate constant (Kep), and the volume transfer constant of the contrast agent (Ktrans) were obtained.

### CT:

The equipment selected was a SOMATOM Definition Flash dual-source CT machine (Siemens, Germany). Firstly, conventional CT scan was performed to determine the target lesion location. The maximum radial section of the solid component of the lesion was selected as the central plane of perfusion scanning. The slice thickness was set to 3-5mm, and four slices were scanned according to the lesion volume. Iodine proll (40ml; Shanghai Bolaike Xinyi Pharmaceutical Co., Ltd., Shanghai, China) and 20ml of normal saline were injected from the superficial vein of the healthy elbow at a rate of 6 ml/s. Perfusion scanning starts with a delay of 20 seconds after injection of contrast medium, and the patient is instructed to hold his breath for 60 seconds. The relevant parameters are set as follows: the layer thickness is 8mm, the visual field is 320mm×320mm, the matrix is 512×512, the current is 100mAs, and the voltage is 80kV. The data were processed and analyzed by Body Pct software, and the average transit time (MTT), product of osmotic surface area (PS) and blood volume (BV) of contrast agent were calculated.

### Collected baseline data and imaging parameters of patients:

- Baseline data included gender, age, disease stage, LNM, degree of differentiation, and pathological type.
- MRI parameters, including ADC, V_e_, K_ep_, K^trans^.
- CT parameters, including MTT, PS, BV.
- The prognosis was divided into complete remission, partial remission, stability, and progression according to the evaluation criteria for solid tumor efficacy. Complete and partial remission were classified as good efficacy, while stability and progression were classified as poor efficacy.

All NSCLC patients were frequently monitored for 18 months (range: 12-26 months at median). Outpatient service and telephone follow-up were conducted every three months. Including clinical symptoms, laboratory tests, CT or MRI scans, and quality of life assessments. Prognostic evaluation was based on solid tumour curative effect criteria. (那此次 研究的数据是治疗前的，还是治疗后?)

### Statistical analysis:

Data analysis was conducted using SPSS version 26.0 software (IBM, Armonk, New York, USA). The measurement data were represented as mean ± standard deviation (SD), and a t-test was used to compare two independent samples between groups. The count data were represented by the number of cases, using the chi-square test. Spearman analysis was used to investigate the correlation between MRI and CT parameters and the physical characteristics of NSCLC. P<0.05 indicated a statistically significant difference.

## RESULTS

There were 62 males and 46 females in the NSCLC group. The group’s age ranged from 49 to 79 years, averaging 63.47 ± 8.13 years; BMI was 16.9~29.2kg/m², with an average of 23.35 ± 2.88kg/m². Disease staging, LNM, differentiation degree, and pathological types of patients are summarized in Table-I.

**Table-I:** Comparison of two groups of baseline data.

Baseline data	NSCLC group (n=108)	Benign group (n=108)	t/χ^2^	P
Male (yes), n(%)	62 (57.41)	59 (54.63)	0.169	0.681
Age (years), mean±SD	63.47±8.13	62.83±9.30	0.537	0.592
BMI(kg/m²), mean±SD	23.35±2.88	22.95±3.02	0.996	0.320
***Disease Staging***				
I + II	51 (47.22)	/	/	/
III+IV	57 (52.78)	/		
***LNM status***				
Yes	47 (43.52)	/	/	/
No	61 (56.48)	/		
***Degree of differentiation***				
Low differentiated	39 (36.11)	/	/	/
Medium to high differentiated	69 (63.89)	/	/	/
***Pathological type***				
Adenocarcinoma	65 (60.19)	/	/	/
Squamous cell carcinoma	43 (39.81)	/	/	/
***Prognosis***				
Good	87 (80.56)	/	/	/
Poor	21 (19.44)	/	/	/

***Note:*** NSCLC = non-small cell lung cancer; BMI = body mass index; LNM = lymph node metastasis.

There were 59 males and 49 females in the benign group, with age ranging from 43 to 79 years and an average age of 62.83 ± 9.30 years. BMI ranged between 16.4 to 28.7kg/m², with an average of 22.95 ± 3.02kg/m². There was no significant difference in baseline data between the two groups (*P*>0.05) (Table-I). The ADC value in the NSCLC group was significantly lower, while V_e_, K_ep_, K^trans^, MTT, PS, and BV were significantly higher than those in the benign group (*P*<0.05) (Table-II).

**Table-II:** Comparison of MRI and CT parameters between NSCLC group and benign group (Mean±SD).

Group	n	MRI parameters				CT parameters		
		ADC (×10^-3^mm2/s)	V_e_	K_ep_ (min)	K^trans^ (min)	MTT (s)	PS (ml/100 mg·min)	BV (ml/100 mg)
NSCLC group	108	1.35±0.33	0.73±0.28	1.65±0.51	3.16±1.06	19.35±4.09	43.14±5.57	10.93±1.43
Benign group	108	1.85±0.54	0.29±0.14	0.73±0.21	1.40±0.36	12.57±1.36	19.05±2.73	4.38±0.95
*t*		-8.145	14.941	17.185	16.300	16.359	40.358	39.665
*P*		<0.001	<0.001	<0.001	<0.001	<0.001	<0.001	<0.001

***Note:*** MRI = magnetic resonance imaging; CT = computed tomography; NSCLC = non-small cell lung cancer.

Among 108 NSCLC patients, patients with stage III+IV, LNM, low differentiation degree, and adenocarcinoma had lower ADC values than those with stage I+II, no LNM, medium to high differentiation, and squamous cell carcinoma. In contrast, V_e_, K_ep_, K^trans^, MTT, PS, and BV were higher in patients with stage I+II, no LNM, moderate to high differentiation, and squamous cell carcinoma (*P*<0.05) (Table-III).

**Table-III:** Comparison of MRI and CT parameters in patients with different pathological features (Mean±SD).

Pathological features	n	MRI parameters				CT parameters		
		ADC (×10^-3^mm2/s)	V_e_	K_ep_ (min)	K^trans^ (min)	MTT (s)	PS (ml/100 mg·min)	BV (ml/100 mg)
***Disease staging***								
Ⅰ+Ⅱ	51	1.49±0.26	0.60±0.23	1.39±0.34	2.80±1.08	17.96±3.49	41.60±5.77	10.30±1.28
Ⅲ+Ⅳ	57	1.23±0.33	0.85±0.26	1.88±0.54	3.47±0.94	20.6±4.20	44.52±5.04	11.50±1.32
*t*		4.565	-5.218	-5.637	-3.434	-3.526	-2.803	-4.794
*P*		<0.001	<0.001	<0.001	0.001	0.001	0.006	<0.001
***LNM***								
No	61	1.46±0.29	0.67±0.25	1.49±0.42	2.89±1.04	18.10±3.49	41.81±5.91	10.62±1.29
Yes	47	1.22±0.33	0.82±0.29	1.85±0.56	3.50±0.99	20.98±4.26	44.86±4.61	11.34±1.51
*t*		3.977	-2.848	-3.813	-3.095	-3.868	-2.920	-2.677
*P*		<0.001	0.005	<0.001	0.003	<0.001	0.004	0.009
***Degree of differentiation***								
Low differentiated	39	1.11±0.29	0.85±0.29	1.97±0.52	3.61±1.01	21.05±4.40	45.39±5.45	11.87±1.19
Medium to high differentiated	69	1.49±0.27	0.67±0.25	1.47±0.42	2.90±1.01	18.39±3.59	41.87±5.26	10.40±1.27
*t*		-6.720	3.332	5.486	3.522	3.408	3.293	5.922
*P*		<0.001	0.001	<0.001	0.001	0.001	0.001	<0.001
***Pathological type***								
Adenocarcinoma	43	1.51±0.29	0.64±0.26	1.46±0.39	2.84±1.11	17.52±3.36	41.30±5.97	10.27±1.24
Squamous cell carcinoma	65	1.25±0.31	0.79±0.27	1.77±0.55	3.36±0.98	20.56±4.10	44.36±4.97	11.36±1.38
*t*		4.496	-2.861	-3.224	-2.557	-4.045	-2.882	-4.167
*P*		<0.001	0.005	0.002	0.012	<0.001	0.005	<0.001

***Note:*** MRI = magnetic resonance imaging; CT = computed tomography; NSCLC = non-small cell lung cancer; LNM = lymph node metastasis.

As shown in Table-IV, ADC values negatively correlated with NSCLC disease staging, LNM, and pathological type. Conversely, there was a positive correlation between ADC values and the degree of differentiation (*P*<0.05). V_e_, K_ep_, K^trans^, MTT, PS, and BV positively correlated with NSCLC disease staging, LNM, and pathological type, and negatively correlated (*P*<0.05) with the degree of differentiation (Table-IV).The ADC values were significantly lower, while V_e_, K_ep_, K^trans^, MTT, PS, and BV were significantly higher in NSCLC patients with poor prognosis compared to patients with good prognosis (*P*<0.05) (Table-V).

**Table-IV:** Correlation Analysis.

Pathological features	ADC		V_e_			K_ep_		K^trans^		MTT		PS		BV	
	r	P	r	P	r		P	r	P	r	P	r	P	r	P
Disease staging	-0.405	<0.001	0.452	<0.001	0.480		<0.001	0.316	0.001	0.324	0.001	0.263	0.006	0.422	<0.001
LNM	-0.360	<0.001	0.267	0.005	0.347		<0.001	0.288	0.003	0.352	<0.001	0.273	0.004	0.252	0.009
Degree of differentiation	0.547	<0.001	-0.308	0.001	-0.470		<0.001	-0.324	0.001	-0.314	0.001	-0.305	0.001	-0.499	<0.001
Pathological type	0.400	<0.001	0.268	0.005	0.299		0.002	0.241	0.012	0.366	<0.001	0.270	0.005	0.375	<0.001

***Note:*** MRI = magnetic resonance imaging; CT = computed tomography; NSCLC = non-small cell lung cancer; LNM = lymph node metastasis.

**Table-V:** Comparison of MRI and CT parameters among patients with different prognosis

Prognosis	n	MRI parameters				CT parameters		
		ADC (×10^-3^mm2/s)	V_e_	K_ep_ (min)	K^trans^ (min)	MTT (s)	PS (ml/100 mg·min)	BV (ml/100 mg)
Poor	21	1.09±0.32	0.98±0.25	2.00±0.64	3.75±0.82	22.79±3.81	45.78±3.74	11.63±1.38
Good	87	1.42±0.30	0.67±0.25	1.56±0.44	3.01±1.06	18.52±3.72	42.50±5.76	10.76±1.39
*t*		-4.501	4.938	3.714	2.977	4.697	2.481	2.568
*P*		<0.001	<0.001	<0.001	0.004	<0.001	0.015	0.012

***Note:*** MRI = magnetic resonance imaging; CT = computed tomography.

## DISCUSSION

This study compared the MRI and CT examination parameters of patients with NSCLC and benign lung diseases. Especially ADC value, PS value and BV value show the highest diagnostic efficiency, which can be used as independent predictors to distinguish NSCLC from benign lung diseases. The ADC value of NSCLC patients decreased significantly, while the values of Ve, Kep, Ktrans, MTT, PS and BV increased significantly. These changes reflected the microenvironment characteristics of NSCLC tissues. In addition, these parameters are significantly related to disease stage, lymph node metastasis, differentiation degree and pathological type, which can provide important reference for clinical evaluation of disease progress and prognosis.

Studies have demonstrated significant differences in the biological behavior, treatment methods, and prognosis evaluation of different pathological types of lung cancer.^5^,^10^ These observations are consistent with previous research. In clinical practice, small cell lung cancer has the characteristics of rapid growth, early systemic metastasis, high responsiveness to specific radiotherapy and chemotherapy, and poor prognosis compared to non-small cell lung cancer.^10^,^11^ Therefore, it is crucial to distinguish the pathological types of lung cancer in clinical practice. Besson et al.^12^ described the advantages of DCE-MRI, which utilizes computational analysis of images before and after injection of contrast agents to obtain relevant examination parameters to implement rapid and continuous imaging based on the circulation of diseased tissue. The diffusion of contrast agents within the lesion tissue may be inferred based on relevant parameters, providing imaging information for diagnosing and evaluating the disease.^12^

A study by Rheinheimer et al.^13^ reported that V_e_, K_ep_, and K^trans^, quantitative parameters obtained through contrast agent diffusion and pharmacokinetic models, are closely related to the degree of blood perfusion and vascular permeability. There is a correlation between V_e_ and extravascular cell density. K^trans^ can reflect the rate at which contrast agents move from the inside of blood vessels to the outside. Its detection value correlates with intercellular space, blood perfusion velocity, and vascular permeability. K_ep_ can reflect the rate of contrast agent movement from intravascular to extravascular loaction.^13^,^14^ Bortolotto et al.^15^ found that DWI mainly detects the diffusion movement of water molecules in tissues, indirectly clarifying the microscopic changes in lung tissue.

The ADC value can accurately reflect the amplitude and range of the diffusion motion. Patients with benign tumors have strong integrity of the vascular basement membrane and limited activity of contrast agents.^13^,^14^ In contrast, NSCLC patients have a more vigorous metabolism of lesion tissue, with an increase in cells and small blood vessels leading to abnormally high vascular permeability and more active contrast agent activity. Therefore, in agreement with our results, V_e_, K_ep_, and K^trans^ values would be higher in patients with benign lesions.^13^-^15^

Han et al.^16^ found a correlation between MRI parameters V_e_, K_ep_, K^trans^ and serum levels of Proliferating Cell Nuclear Antigen (PCNA), Survivor, and Vascular endothelial growth factors (VEGF) in NSCLC patients, which has certain reference value for the assessment of patient condition and prognosis evaluation. The results of this study further support this observation. Additionally, a study by Zhang et al.^17^ confirmed that DCE-MRI and DWI examination parameters can be used to evaluate the early efficacy of NSCLC after radiotherapy and chemotherapy. Moreover, it can provide information on the internal environment of tumor tissue and effectively reflect the changes in tumor cell structure and microcirculation status.

This study demonstrated significant differences in MTT, PS, and BV among NSCLC patients with different disease stages, LNM status, differentiation degree, pathological type, and prognosis. This is consistent with previous research findings.^18^,^19^ Liu et al.^18^ pointed out that the pathological basis for the multi-slice computed tomography (MSCT) examination is significant differences in the microcirculation status and angiogenesis between benign nodules and malignant lesions in the lungs. BV can reflect the blood volume of the lesion and is related to the number and diameter of blood vessels. PS is closely related to the permeability of capillary endothelial cells.^19^,^20^ The main reason for the significant differences in MTT, PS, BV, etc. between patients with NSCLC and benign tumor lesions is that the growth of benign tumor lesions is slower.

Moreover, NSCLC can lead to the formation of many new blood vessels under the induction of vascular growth factors, coupled with significantly increased microvascular permeability, making it easy for contrast agents to diffuse into the interstitial tissue.^19^–^21^ This confirms that MRI and CT examinations have high application value in the diagnosis and treatment evaluation of NSCLC, can clarify the pathological characteristics of the disease, evaluate the treatment status, and provide a reference for the formulation or adjustment of follow-up intervention plans for NSCLC.

### Limitations

First, it is a retrospective study with a small sample size. Second, relatively few pathological types of lung cancer were included, lacking cases such as large cell carcinoma and carcinoid carcinoma. Third, this study only discussed solid nodules and did not explore subsolid nodules and ground glass nodules. This exclusion criterion limits the generalization of research results to all NSCLC cases, especially as early lung cancer typically has ground glass nodules. To increase the use of MRI and CT parameters in NSCLC diagnosis and prognosis, future research should determine the best imaging parameters and measuring methodologies for these lesions. Finally, imaging parameters may be influenced by human or technical factors, and manually delineating regions of interest may introduce certain variability in the results due to errors and subjective factors.

## CONCLUSION

In this study the diagnostic and prognostic value of MRI and CT parameters in non-small cell lung cancer was systematically evaluated. ADC, PS and BV parameters show high diagnostic efficiency, which can effectively distinguish non-small cell lung cancer from benign lesions, and are significantly related to disease stage, lymph node metastasis and differentiation. These imaging parameters can provide an objective basis for the diagnosis, prognosis evaluation and treatment monitoring of non-small cell lung cancer.

### Authors’ contributions:

**LB:** Study design, literature search, manuscript writing, manuscript revision validation and critical analysis.

**JW, ZZ and YZ:** Data collection, data analysis and interpretation. Critical review.

All authors have read, approved the final manuscript and are responsible for the integrity of the study.
